# Short-term outcomes of transcatheter perimembranous ventricular septal defect closure using the konar-multifunctional occluder: the Taiwanese experience

**DOI:** 10.3389/fcvm.2025.1572812

**Published:** 2025-05-27

**Authors:** Li-Chin Liao, Yun-Ching Fu, Pi-Chang Lee, Sheng-Ling Jan, Ming-Chih Lin, Chieh-Mao Chuang, Hui-Chih Hung

**Affiliations:** ^1^ Doctoral Program in Translational Medicine, National Chung Hsing University, Taichung, Taiwan; ^2^ Rong Hsing Translational Medicine Research Center, National Chung Hsing University, Taichung, Taiwan; ^3^ Department of Pediatrics, Wuri Lin Shin Hospital, Taichung, Taiwan; ^4^ Department of Pediatric Cardiology, Children’s Medical Center, Taichung Veterans General Hospital, Taichung, Taiwan; ^5^ Department of Pediatrics, School of Medicine, National Chung Hsing University, Taichung, Taiwan; ^6^ Department of Pediatrics and Institute of Clinical Medicine, National Yang Ming Chiao Tung University, Taipei, Taiwan; ^7^ Department of Post-Baccalaureate Medicine, College of Medicine, National Chung Hsing University, Taichung, Taiwan; ^8^ Department of Life Sciences, National Chung Hsing University, Taichung, Taiwan; ^9^ iEGG & Animal Biotechnology Center, National Chung Hsing University, Taichung, Taiwan

**Keywords:** ventricular septal defect, occluder, transcatheter closure, KONAR-MF occluder, congenital heart defect

## Abstract

**Introduction:**

Transcatheter device closure of perimembranous ventricular septal defect (PmVSD) using the Lifetech KONAR-MF ventricular septal defect occluder (MFO) presents a promising and effective alternative to surgical repair.

**Objectives:**

This study aims to evaluate the 6-month safety and efficacy of the MFO device for PmVSD closure.

**Materials and methods:**

We conducted a retrospective analysis of clinical data from patients who underwent percutaneous PmVSD closure using the MFO device at our institution between December 2021 and June 2024. Safety, procedural and 6-month outcomes were systematically assessed.

**Results:**

A total of 115 patients (52.2% male) underwent transcatheter PmVSD closure. The median age was 7.6 years [interquartile range (IQR), 4.0–27.2] and weight 25.6 kg (IQR, 14.2–62.6). Median defect size by angiography was 5.7 mm (IQR, 3.8–8.3) on the left ventricle side and 3.3 mm (IQR, 2.3–4.4) on the right ventricle side. Aortic valve prolapse (AVP) was noted in 114 patients (99.1%), with pre-procedural aortic regurgitation (AR) in 36 (31.3%). Median pulmonary artery pressure was 17 mmHg (IQR, 14–20); 48 (41.7%) had Qp/Qs >1.5. All procedures were successful; 33 (28.7%) used a retrograde approach. Median fluoroscopy time was 22 min (IQR, 15–33). Complete closure was achieved in 51.3% at 1 day, 62.6% at 1 month, 69.6% at 3 months, and 83.5% at 6 months. Transient conduction disturbances (*n* = 4), hypotension (*n* = 1), and femoral hematoma (*n* = 1) were observed. No cases of endocarditis, valve injury, or complete atrioventricular block occurred.

**Conclusion:**

Transcatheter closure of PmVSD with the MFO demonstrated safety and efficacy during the 6-month follow-up period. Notably, the majority of defects in this cohort were small in size.

## Introduction

Perimembranous ventricular septal defect (PmVSD) is the most common congenital heart disease (1). Since Lock et al. first achieved a successful percutaneous ventricular septal defect (VSD) closure in 1988, transcatheter approaches have been recognized as a viable treatment option in selected patients. Progress in device design and procedural methods has allowed this intervention to be performed regularly in many healthcare institutions (2, 3). Early approaches primarily utilized double-disc devices, which required sufficient septal and aortic rims, making them unsuitable for many defects (4, 5). The Amplatzer asymmetrical membranous VSD occluder (St. Jude Medical, St. Paul, MN, USA) was specifically engineered for PmVSD and demonstrated generally favorable outcomes with widespread use (6, 7). However, its association with a significant risk of complete atrioventricular (AV) block led to its discontinuation (3, 8). Currently, devices such as the Nit-Occlud® Lê VSD-Coil (PFM Medical, Germany) (9), Cera VSD devices (Lifetech, Shenzhen, China), and other device occluders from Amplatzer (Abbott, USA) are being utilized (8, 10). In a meta-analysis comparing percutaneous and surgical closure of PmVSD, both approaches demonstrated comparable procedural success, major complications, and valvular outcomes, though percutaneous closure offered advantages in terms of shorter hospital stay and lower transfusion requirements (11). Despite ongoing debates and limited regulatory approval in several countries, transcatheter closure of PmVSD continues, including in Taiwan, where various adapted devices, initially intended for different conditions, are used off-label. PmVSD closure remains challenging due to anatomical complexities, such as proximity to the aortic and tricuspid valves, right coronary cusp (RCC) prolapses, and the risk of complete heart block. An optimal device for this procedure has yet to be developed. The Lifetech KONAR-MF ventricular septal defect occluder (MFO), a CE-approved for VSD closure, flexible device with single- and double- disc features, that allows antegrade and retrograde delivery, minimizing structural damage and complications; The MFO has been increasingly adopted in recent years for PmVSD closure due to its smart features, and data on its safety and efficacy in this population is accumulating (12, 13). Despite its growing use worldwide, clinical data, particularly from East Asia, remain scarce. This study provides short-term real-world data on the MFO device for PmVSD closure in Taiwan, focusing on procedural safety, efficacy, and anatomical considerations.

## Methods

### Study design and population

We conducted a retrospective analysis of clinical data from patients who underwent percutaneous PmVSD closure using the MFO device at our institution between December 2021 and June 2024. Safety, procedural and 6-month outcomes were systematically assessed. Each case was approved by a multidisciplinary team, and patients were informed of all treatment options, including surgery. The study complied with local regulations and institutional requirements, with protocol approval from the institutional review board [IRB TCVGH No. CG16272B]. Written informed consent was obtained from participants' legal guardians or next of kin.

Inclusion criteria: Patients with clinically symptoms and hemodynamically relevant PmVSD were included in this study. Hemodynamically significant VSDs were identified in patients with left heart chamber enlargement, evidence of pulmonary hypertension, a pulmonary-to-systemic flow ratio (Qp/Qs) exceeding 1.5, cardiomegaly on chest radiography, or clinical manifestations such as frequent respiratory infections, failure to thrive, or weight loss. Patients with PmVSD and mild RCC prolapse were eligible only if aortic regurgitation (AR) was assessed as trivial or mild.

Exclusion criteria: Patients with PmVSD were excluded if they had more than mild aortic valve regurgitation or prolapse, severe pulmonary hypertension [mean pulmonary artery pressure (mPAP) ≥ 35 mmHg], other congenital heart defects requiring surgery, or large VSDs [right ventricle [RV]-side diameter > 14 mm or left ventricle [LV]-side diameter > 18 mm] requiring an MFO device size > 14/12. Additionally, patients with a body weight < 7 kg, active bacterial infections (e.g., endocarditis or sepsis), or malaligned VSDs were excluded.

### AR assessment

AR was evaluated in all patients using transthoracic echocardiography (TTE) by experienced pediatric cardiologists. The severity of regurgitation was graded according to the guidelines of the American Society of Echocardiography (ASE), using an integrative approach based on multiple parameters, including jet width, vena contracta width, and the presence of holodiastolic flow reversal in the descending aorta.

### Study device and device selection protocol

The MFO is a self-expanding, double-disc device made of 144 nitinol wires connected by a cone-shaped waist. It comes in eight sizes (5/3 to 14/12 mm); larger sizes (≥9/7 mm) are reinforced with a polytetrafluoroethylene (PTFE) membrane to improve occlusion, while smaller ones are not. The device is delivered via the SteerEase™ introducer (Lifetech, Shenzhen, China), using 5F to 7F sheaths depending on the device size. Device selection followed previously published recommendations (14). Conical defects (RV/LV ratio ≤ 0.5) were closed with waist diameters 1–2 mm larger than the LV size, while tubular defects (RV/LV > 0.5) were oversized by 2–3 mm. For subaortic rims ≥ 2 mm, the right disc (D2) was chosen 1–2 mm larger than the LV opening; for deficient rims (<2 mm), D2 matched the LV size.

### Study procedure

The procedure was performed under local anesthesia or conscious sedation with TTE guidance. Femoral vein and artery access were obtained, and patients received heparin (75–100 IU/kg) and antibiotics prior to the intervention. Left ventriculography was performed to assess the defect size, morphology, and relationships to surrounding structures. Device size was selected based on measurements at the narrowest right-side diameter of the defect, with adjustments for aneurysms or deficient aortic rims. The approach, retrograde or antegrade, was chosen based on the patient's anatomy and procedural requirements. All procedures were performed under conscious sedation via femoral access. An arteriovenous loop (AVL) was routinely established at the beginning of each procedure to enable antegrade delivery of the device. This approach was preferred due to the ability of the femoral vein to accommodate larger sheath sizes and was especially suitable in younger or smaller patients, as well as in cases where the defect was located close to the tricuspid valve or exhibited a complex aneurysmal morphology. The retrograde approach was utilized selectively when advancement of the AVL was unsuccessful, most commonly due to entanglement with the tricuspid valve or difficulty navigating through elongated aneurysmal tissue. In such cases, direct retrograde delivery from the femoral artery allowed for a more favorable trajectory and simplified device positioning. Deployment of the device involved careful positioning of the left and right discs to ensure no interference with valve function. TTE and angiography confirmed proper placement and closure before release. Post-procedure, patients received aspirin (3–5 mg/kg daily for 3 months) if there was no residual shunt and were followed up with echocardiography at regular intervals to monitor outcomes and assess residual shunts or valve complications.

### Post-operative care and follow-up

Postprocedural anticoagulation with a 24 h Heparin infusion was deemed unnecessary in all patients. In the absence of a residual shunt, patients received oral aspirin (3–5 mg/kg daily) for 3 months. Follow-up assessments, including physical examination, TTE, and electrocardiography (ECG), were performed at 1 day, 1, 3, and 6 months. Residual shunts were categorized by Doppler jet width: trivial (<1 mm), small (1–2 mm), moderate (3–4 mm), and large (≥4 mm). Valve regurgitation and rhythm disturbances were evaluated based on new or persistent complications requiring intervention.

### Statistical analysis

Categorical variables were presented as frequencies and percentages, while continuous variables were reported as mean ± standard deviation or median with interquartile range (IQR), based on data distribution assessed by the Shapiro–Wilk test. Group comparisons were conducted using the chi- squared test for categorical variables and Student's *t*-test for continuous variables, with statistical significance set at two-sided *p*-value < 0.05.

## Results

### Patients

Transcatheter closure of PmVSD using the MFO device was attempted in 115 patients. Sixty (52.2%) were male. The median age was 7.6 years (IQR, 4–27.2), and the median body weight was 25.6 kg (IQR, 14.2–62.6) (Table 1).

**Table 1:** Demographic characteristics of the patients.

Total (*n* = 115)	
Variable	Number (%) or median [IQR]
Age (Months)	7.6 (4.0–27.2)
Body weight (kg)	25.6 (14.2–62.6)
Sex (Male)	60 (52.2%)
With septal aneurysm	107 (93.0%)
Size of VSD (mm)	
LV side (TTE), mm	5.5 (4.6–6.7)
RV side (TTE), mm	4.1 (2.9–4.8)
LV side (Angiography), mm	5.7 (3.8–8.3)
RV side (Angiography), mm	3.3 (2.3–4.4)
Size distribution of VSD, LV side (mm)	
<4	33 (28.7%)
4–8 mm	49 (42.6%)
>8	33 (28.7%)
Mild aortic valve prolapse	114 (99.1%)
Mitral valve insufficiency	
Absent	56 (48.7%)
Trivial	19 (16.5%)
Mild	40 (34.8%)
Moderate	0
Tricuspid valve insufficiency	
Absent	76 (66.1%)
Trivial	16 (13.9%)
Mild	15 (13.0%)
Moderate	8 (7.0%)
Aortic valve insufficiency	
Absent	79 (68.7%)
Trivial	21 (18.3%)
Mild	15 (13.0%)
Moderate	0

VSD, ventricular septal defect; LV, left ventricle; RV, right ventricle; TTE, transthoracic echocardiography.

[Data are presented as number (%) or as median [interquartile range, IQR].

### Defects

On TTE, the median diameter of the VSD on the LV side was 5.5 mm (IQR, 4.6–6.7), and 4.1 mm (IQR, 2.9–4.8) on the RV side. On angiography, the median LV-side diameter was 5.7 mm (IQR, 3.8–8.3), and the RV-side diameter was 3.3 mm (IQR, 2.3–4.4). Aneurysmal tissue of the membranous septum was identified in 107 (93.0%) patients. Mild AVP was present in 114 (99.1%) patients—111 RCC and 3 non-coronary cusp (NCC) prolapse—with pre-procedural AR in 36 patients (21 trivial, 15 mild) (Table 1).

### Procedure

**Figure 1:**
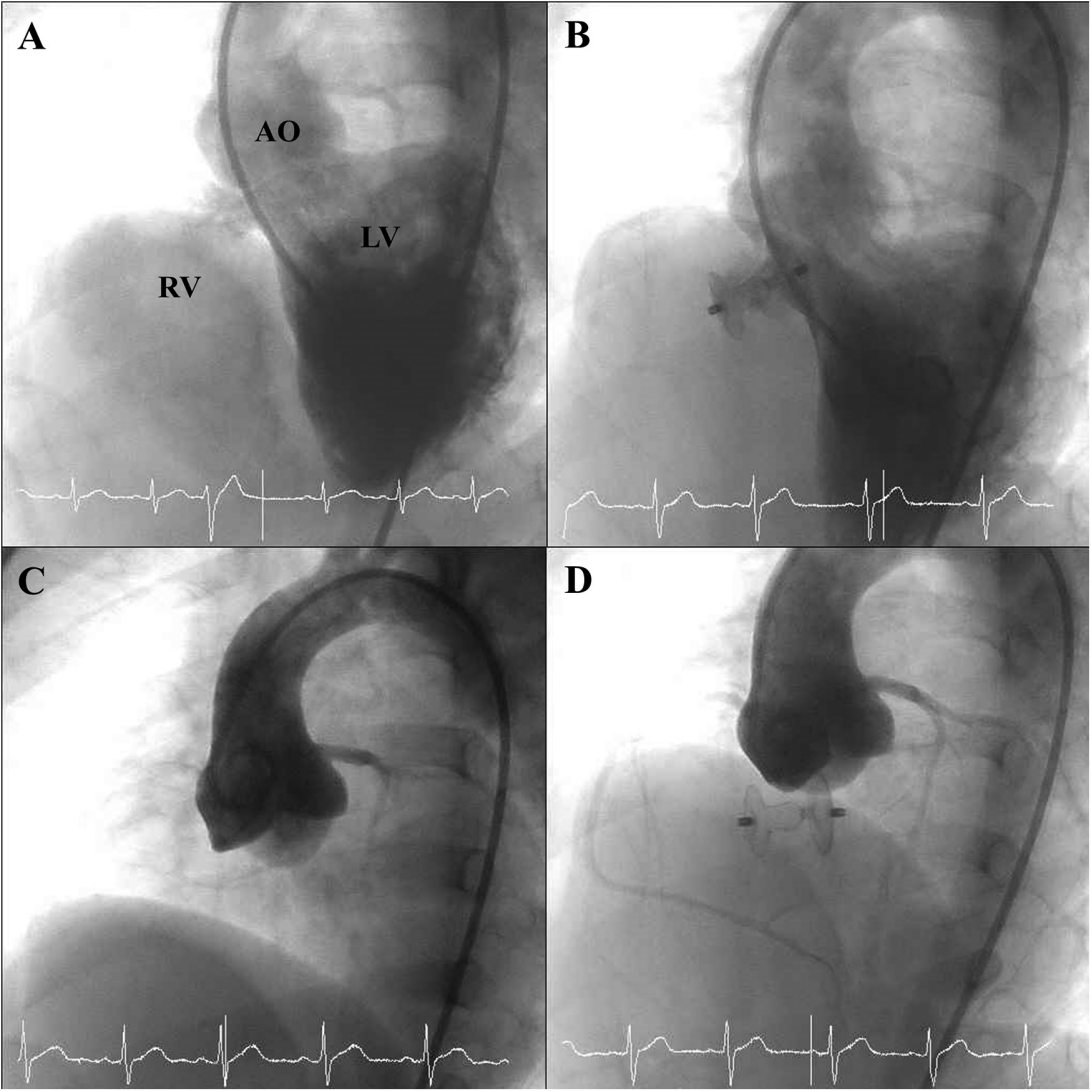
Angiographic findings before and after device deployment. **(A)** Pre-deployment angiographic image illustrating aneurysmal tissue of the membranous septum associated with the PmVSD. **(B)** Post-deployment angiographic view showing successful closure of the PmVSD without residual shunt. **(C)** Pre-deployment angiographic view demonstrating AVP associated with a PmVSD. **(D)** Post-deployment angiographic view showing persistent AVP in relation to the PmVSD, without interference from the occluder device.

Closure indications included abundant shunting with Qp/Qs >1.5 (*n* = 48, 41.7%), mild AVP (*n* = 114), and failure to thrive (*n* = 71, 61.7%). Some had multiple indications. The median Qp/Qs ratio was 1.36 (IQR, 1.2–1.7), and the median mPAP was 17 mmHg (IQR, 14–20). Angiographic assessment before device implantation demonstrated PmVSD frequently accompanied by aneurysmal transformation of the membranous septum (Figure 1A) and, in most cases, mild AVP (Figure 1C). The median device size was 8 × 6 mm (range, 5 × 3 to 14 × 12 mm). The antegrade approach was initially attempted in 82 (71.3%) procedures and the retrograde approach in 33 (28.7%), with crossover in 11 procedures. The median procedure time was 62 min (IQR, 52–84), and the median fluoroscopy time was 22 min (IQR, 15–33) (Table 2). Post-deployment angiography confirmed successful device placement without interference with aortic valve function (Figure 1D), and no residual shunt was observed in the majority of cases (Figure 1B).

**Table 2:** Procedural features.

Total (*n* = 115)	
Variable	Number (%) or median [IQR]
Pulmonary arterial hypertension	
No	87 (75.7%)
Mild (21–30 mmHg)	24 (20.9%)
Moderate (31–45 mmHg)	4 (3.5%)
PAm (mmHg)	17 (14–20)
Qp/Qs	1.36 (1.2–1.7)
Approach used for device closure	
Antegrade	82 (71.3%)
Retrograde	33 (28.7%)
Implanted device size (mm)	
5 × 3	6 (5.2%)
6 × 4	32 (27.8%)
7 × 5	6 (5.2%)
8 × 6	40 (34.8%)
9 × 7	5 (4.3%)
10 × 8	20 (17.4%)
12 × 10	3 (2.6%)
14 × 12	3 (2.6%)
Device characteristic	
Without PTFE membrane	84 (73%)
With PTFE membrane	31 (27%)
Duration of procedure (min)	62 (52–84)
Fluoroscopy time (min)	22 (15–33)

Pam, mean pulmonary arterial pressure; PTFE, polytetrafluoroethylene; Qp, pulmonary blood; Qs, systemic blood flow.

[Data are presented as number (%) or as median [interquartile range, IQR].

### Post-operative care and complications

Percutaneous device closure was successful in all patients. No major vascular complications, mortality, device embolization, infective endocarditis, erosion, or thromboembolic events occurred. Four patients experienced transient arrhythmias (1 s-degree AV block, 2 right bundle branch block, 1 left bundle branch block), all of which resolved after steroid therapy. One patient had transient hypotension, and another developed a femoral hematoma. During the 6-month follow-up, no additional complications, including arrhythmias, valve dysfunction, or device-related issues, were noted (Table 3).

**Table 3:** Outcomes, follow up, complications related to procedure.

Total (*n* = 115)	
Variable	Number (%) or median [IQR]
Procedural success	
Successful closure	115 (100%)
Complete occlusion of shunt	
1-day follow-up	59 (51.3%)
1-month follow-up	72 (62.6%)
3-month follow-up	80 (69.6%)
6-month follow-up	96 (83.5%)
Complications	
Major	
Device embolization	0
Increase in aortic regurgitation	0
Minor	
Transient hypotension	1 (0.9%)
Hematoma	1 (0.9%)
Heart block/AV block	
RBBB/LBBB	2 (1.7%)
RBBB/LBBB	1 (0.9%)
Second-degree AV block	1 (0.9%)

MFO, lifetech konar-MF VSD occluder; RBBB, right bundle branch block; LBBB, left bundle branch block; AV, atrioventricular.

[Data are presented as number (%) or as median [interquartile range, IQR].

### Follow-up

**Figure 2:**
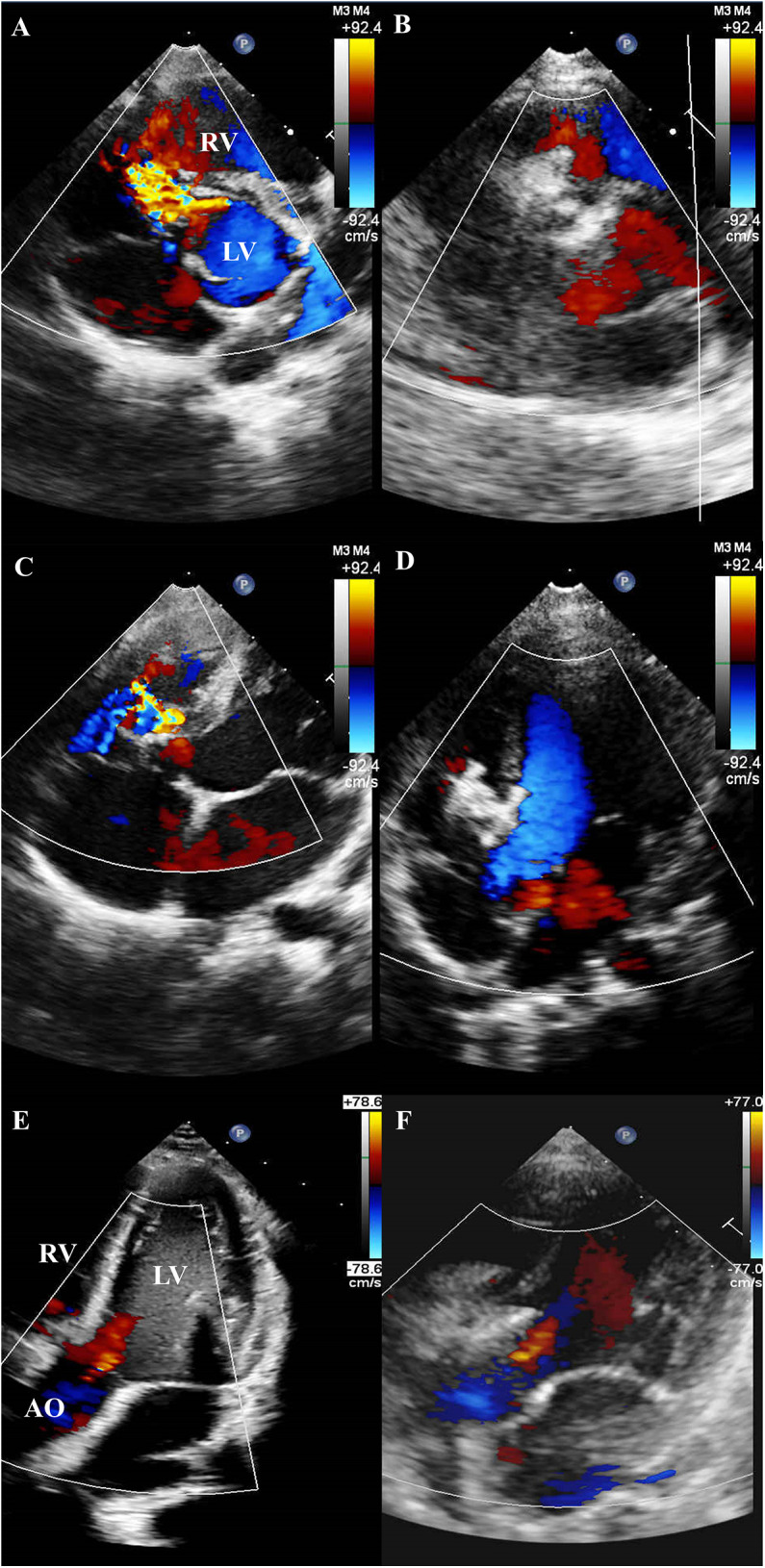
TTE findings before and after device implantation. **(A)** Pre-procedural TTE (short-axis view) revealing a PmVSD with associated aneurysmal tissue. **(B)** Follow-up TTE at 6 months (short-axis view) demonstrating complete closure of the PmVSD without residual shunt. **(C)** Pre-procedural TTE revealing a PmVSD with associated aneurysmal tissue. **(D)** Follow-up TTE at 6 months demonstrating complete closure of the PmVSD without residual shunt. **(E)** Pre-procedural TTE demonstrating mild AR. **(F)** Follow-up TTE at 6 months showing persistent mild AR, with no evidence of progression following device implantation.

Residual shunts were detected in 57 (49.6%) patients after device deployment—17 trivial, 32 mild, and 8 moderate. Complete closure rates improved progressively over time: 51.3% the next day, 62.6% at 1 month, 69.6% at 3 months, and 83.5% at 6 months. No patient developed progressive AR during follow-up. TTE further illustrated the structural changes before and after intervention. Pre-procedural TTE revealed aneurysmal tissue in the short-axis view (Figure 2A) and mild AR in several cases (Figure 2C). At 6-month follow-up, TTE confirmed complete closure of the defect without residual shunting (Figure 2B), and the degree of AR remained stable in all patients, with no evidence of progression (Figure 2D).

## Discussion

Our study achieved a 100% procedural success rate and an 83.5% complete closure rate. There were no cases of hemolysis, permanent heart block, endocarditis, or other valvar complications during short-term follow-up, and no fatalities were reported, further supporting the MFO device's safety profile. Compared to the MIOS-MFO multicenter study (*n* = 333) (15), our cohort (*n* = 115) showed a lower 6-month complete closure rate (83.5% vs. 97.1%) but no cases of device embolization (0% vs. 2.4%). This may be attributed to our strict inclusion policy favoring small defects, which are potentially less prone to complications such as device embolization. However, proper case selection, careful consideration of identified risk factors, and appropriate device matching are crucial, as embolization may occur, immediately and during early follow-up (16). Smaller cohort studies in the literature also reported low embolization rates with the MFO device: Tanıdır et al. (13) reported a 2%, and Haddad et al. (12) described one case occurring 24 h post-procedure. In our series, device migration into the RV occurred intra-procedurally in two patients (4.1%), both of whom presented with high-risk anatomical features (e.g., defect location or an aortic rim <2 mm). Contributing factors likely included underestimation of defect size, suboptimal device selection, or technical challenges during deployment. Moreover, the MFO's dual delivery design enhances procedural flexibility, allowing both antegrade and retrograde approaches (17). However, in our study, the retrograde route was used in only 28.4% of cases, primarily due to sheath size limitations in smaller children.

Importantly, the markedly higher prevalence of mild AVP in our cohort (99.1% vs. 10.5%) likely reflects differences in clinical presentation, timing of diagnosis, and intervention, as well as broader case selection criteria driven by national health insurance reimbursement policies. However, prior studies have also demonstrated successful pmVSD closure in patients with AVP and less than mild aortic regurgitation using various devices, including the MFO (7, 15, 18).

### Residual shunt

Residual shunting rates reported in the literature vary. In a comprehensive study by Haddad et al. (12), complete closure rate of 40% (8/20) at the end of the procedure, which increased to 57.9% (11/19) at discharge and 84.2% (16/19) after six months of follow-up. In our study, immediate post- procedural closure was relatively lower, with fewer than 25% of patients exhibiting small, smoky residual shunts. This may reflect a delayed closure process, as the smaller MFO sizes (first four models) lack a PTFE membrane that would otherwise promote thrombosis. These residual flows were not hemodynamically significant and were thus considered acceptable. Nevertheless, both prior studies and our current investigation confirm that residual shunts tend to decrease over time (12, 13).

### Heart block

Complete AV block after transcatheter pmVSD closure is typically observed either early in the procedure or later during short-term follow-up, which has limited the widespread adoption of this method in several countries (3, 6, 7, 19). Oversized devices may cause early-onset heart block due to clamping force and mechanical trauma during deployment, while late-onset heart block can result from fibrosis, compression, or inflammation of the conduction system (20). Smaller infants are also considered at higher risk of developing heart block following percutaneous pmVSD closure (19). Unlike some conventional pmVSD occluders that require large-profile delivery systems, the MFO can be deployed using a smaller 4–7 Fr sheath or guiding catheter (21). In comparison with published data, Tanıdır et al. (13) reported transient AV block in two patients, both of which resolved after sheath withdrawal without the need for pacemaker implantation. Haddad et al. (12), as well as the MIOS-MFO multicenter study (15), reported no rhythm disturbances in their respective small patient cohorts. Nevertheless, heart block may still occur in rare cases, particularly in anatomically challenging defects or suboptimal device selection (13, 22), underscoring the importance of long-term rhythm monitoring until this risk is fully mitigated. In our study, despite a younger and lower-weight cohort, only one case of transient AV block was observed, which was resolved with medical therapy. Notably, most defects in our series were small and associated with membranous septal aneurysms—an anatomic feature previously suggested to be protective against conduction disturbances.

### Valvar disturbances

Aortic and tricuspid regurgitation are potential complications that are often closely monitored during the procedure. Haddad et al. (12) emphasized the continuous assessment of the right disk position via echocardiography. Their study found no instances of tricuspid regurgitation either at the conclusion of the procedure or during follow-up. Similarly, Tanıdır et al. (13) reported only one patient of moderate tricuspid insufficiency (1%) in their series.

New-onset AR following transcatheter closure of PmVSD has been reported in up to 16% of patients with ductal occluders (23), and up to 17% with the Amplatzer membranous occluder (3, 6, 12, 19). The incidence of new-onset AR is lower with the ADO II (24) and the modified Chinese occluder (25, 26).

Regarding aortic valve insufficiency, only one patient experienced it in the study by Haddad et al. (12), and mild AR was noted in one patient in the research by Tanıdır et al. (13). In our study, only two cases of trivial AR occurred, without progression during follow-up. Notably, all AVP cases in this study were mild, with no progression to moderate or severe AR during follow-up, suggesting that transcatheter closure with the MFO device is safe and feasible in carefully selected patients. These findings also support that mild AVP should not be an absolute contraindication to device closure, though prior research highlights valve prolapse and subaortic rim deficiency as risk factors for post-procedural valve dysfunction (15, 27). However, we believe the device's soft, flexible design with a nitinol wire mesh layer allows it to conform to the plane of the aortic valve without disrupting leaflet coaptation.

### Study limitations

This study has several limitations. In addition to its single-center, retrospective design, the six-month follow-up may be insufficient to detect delayed complications such as progressive aortic regurgitation or conduction abnormalities. The predominance of small defects in our cohort further limits the generalizability of these findings to larger or more complex anatomies. Moreover, selection bias may have contributed to the high prevalence of AVP, as Taiwan's National Health Insurance reimburses pmVSD closure only for patients with clinically significant defects—such as cardiomegaly, heart failure symptoms, or AVP without severe aortic regurgitation—excluding those with small, asymptomatic shunts.

## Conclusion

Based on our short-term results, transcatheter closure of PmVSD using the MFO device is both feasible and safe. Most defects in this cohort were relatively small, which should be taken into account when interpreting the outcomes.

## Data Availability

The original contributions presented in the study are included in the article/Supplementary Material, further inquiries can be directed to the corresponding author/s.
